# Estimation and Prediction of Vertical Deformations of Random Surfaces, Applying the Total Least Squares Collocation Method

**DOI:** 10.3390/s20143913

**Published:** 2020-07-14

**Authors:** Zbigniew Wiśniewski, Waldemar Kamiński

**Affiliations:** 1 Faculty of Geoengineering, University of Warmia and Mazury in Olsztyn, 10-957 Olsztyn, Poland; zbyszekw@uwm.edu.pl; 2 Faculty of Civil and Environmental Engineering, Gdańsk University of Technology, 80-233 Gdańsk, Poland

**Keywords:** deformation analysis, free control network, collocation, total least squares

## Abstract

This paper proposes a method for determining the vertical deformations treated as random fields. It is assumed that the monitored surfaces are subject not only to deterministic deformations, but also to random fluctuations. Furthermore, the existence of random noise coming from surface’s vibrations is also assumed. Such noise disturbs the deformation’s functional models. Surface monitoring with the use of the geodetic levelling network of a free control network class is carried out. Assuming that, in some cases, the control networks are insufficient in surface’s deformation analysis, additional and non–measurable reference points have been provided. The prediction of these points’ displacements and estimation of the free control network points’ displacement are carried out using the collocation method applying the total least squares adjustment. The proposed theoretical solutions were verified by the simulation methods and on the example of a real control network.

## 1. Introduction

Engineering structures require monitoring and estimation of their technical conditions. Various systems and measurement techniques are used for this purpose, for example, those constructed with the use of specialized sensors and detectors [1,2]. Geodetic methods used for accurate determination of positions of monitored structure selected points have a significant role in the stability monitoring of buildings, bridges, and dams, among others. Geodetic monitoring is conducted using a variety of techniques and measurement methods. The most common are global navigation satellite systems [3,4,5], photogrammetry, and remote sensing [6], as well as laser scanning [7]. However, the classic geodetic control networks [8,9] still play a basic role in the engineering structures’ deformation analysis with the use of the geodetic methods. Structural deformation is then determined on the basis of changes of points’ positioning (displacements) within this network over time. Such changes are determined on the basis of periodic measurements (measurement epochs).

Geodetic control networks are comprised of controlled points and reference points. Reference points require a stability analysis carried out applying appropriate procedures and statistical tests [10,11]. On the basis of the networks measurements conducted in two or more epochs, the displacements of controlled points and potential reference points are then determined. The displacements of these points can be obtained by determining the differences between the estimators of the networks coordinates in different measurement epochs. The displacements can be also treated as parameters in models of the observations differences [12,13]. In this case, in the literature of the subject, the displacement of individual points of the network is sometimes treated as the shift of the model’s parameters [14,15]. The resignation from the reference points leads to solutions that apply the principles of free adjustment [16,17,18,19]. Such networks belong to the free control networks (FCNs) class. The principles of free adjustment [16,17,19,20] are applied to determine these networks points’ displacements. These principles derive from the free adjustment theory presented in numerous papers [21,22,23,24,25].

It is generally assumed that the deformation of a structure in time is deterministic. There are, however, structures for which this assumption is inadequate, particularly if they are situated in a dynamically changing environment or are a subject to dynamic loads [26]. For example, when analysing the bridges’ deformations, not only deterministic deformations can be considered, but also the deformations caused by vehicles traffic and other factors as well as random strains and vibrations of the examined object [27,28]. This also applies to such structures as river dams, tunnels, port quays, and so on [29,30]. In many cases, the object of interest is the deformation of selected surfaces of engineering structures. The results of the object’s random deformations are the fluctuations of its surface, whereas the result of the vibration is the random noise. In this approach, the monitored surfaces can be treated as random fields represented by appropriate random functions.

In practice, there are situations when the subject of interest is focused on the positioning changes not only of the controlled points (CPs), but also of the additional points that are not directly related to the geodetic network. These points, hereinafter called the extended controlled points (ECPs), are necessary for examining the engineering object condition. However, it is not possible to determine their displacements based on the direct geodetic observations (e.g., covering, lack of access, damage). It is assumed that ECPs are situated in close proximity to actual controlled points. Assuming that the monitored surface is a random field, then the use of statistical interpolation and prediction methods could be proposed to determine ECPs’ displacement. It is important to remember that there are limited possibilities of using ECPs determined in this way. However, in certain cases, the solutions proposed below can be the way to approach this issue (or to complete other methods) in difficult and unconventional situations.

The least-squares collocation (LSC) is the most popular method of interpolating and predicting the values of random functions measured in discrete points. The idea of this method was proposed and developed in the following studies inter alia [31,32,33,34,35]. The LSC was applied in solving various geodetic problems, including, inter alia, determination of gravimetric measurements results [36,37,38] and coordinates transformation [39,40,41,42]. In the paper [43], the use of collocations in the mobile LiDAR (Light Detection and Ranging) system was proposed. Tscherning [44,45] applied the LSC method in analysing the observations gathered during the GOCE (Gravity field and steady-state Ocean Circulation Explorer) mission. The principles of this collocation were also the basis of the analysis of deformations caused by earthquakes [46]. Yang [47] presented a way for the robustness of the collocation method against the observations’ gross errors.

In this paper, it is assumed that FCN is a levelling network. All of its points serve as CPs and are located on a surface that is characterised by deterministic and random deformations. The general LSC principles are applied in determining the vertical displacements of CPs as well as in displacements of non-measurable ECPs. In the paper, it will be presented that surface noise leads to random disturbances of the special matrices occurring in functional observation models. For this reason, the conventional solutions used in LSC will be replaced by solutions proposed in the total least squares (TLS) theory. The theory and basic applications of TLS were discussed, inter alia, in [48,49,50]. The collocation that uses the TLS for solving the optimisation problem is called TLSC (total least-squares collocation). The application of TLSC will allow not only the determination of the deterministic and random displacements of CPs and ECPs, but also the estimation of the value of random disturbance in these points.

## 2. Basic Assumptions and Models

### 2.1. Surface Fluctuation

Assume that the surface π is the subject to the time-variable t∈T vertical displacements. These deformations are expressed by changes in the heights H(t) of its points (in the adopted reference system). Assume now that vertical deformations are subject to time- and position-dependent random fluctuations. When fluctuations are described by the random function $\xi_{t}(\omega )$, where ω∈Ω is the position parameter (e.g.,ω=(x,y)), then the heights of the points on the surface can be expressed as

(1) $$ℋ(t,\omega )=H(t)+\xi_{t}(\omega )$$

For function $\xi_{t}(\omega )$, it is assumed that $E\{\xi_{t}(\omega )\}=0$, hence E{ℋ(t,ω)}=H(t), where E(∘) is an expected value. In the simplest case, the fluctuations can be treated as signals $s_{t}(\omega )$ with assumed covariance function. In this case, $\xi_{t}(\omega )=s_{t}(\omega )$. This approach is applied in solving physical geodesy problems, for instance, in processes of filtering and predicting the gravimetric anomalies [32,33,38].

When surface π is an element of the engineering object, then not only its deterministic deformations and random fluctuations, but also additional noise caused by structural vibrations [28,29], should be projected. This noise should be marked with $e_{t}$ and called the primary surface noise. Assume that these are random values with an expected value of $E(e_{t})=0$ and variance of $\sigma_{e}^{2}$ (for each t∈T). Furthermore, similarly to [51], it is assumed that surface noise is Gaussian.

At each point $P_{f}\in \pi$ with coordinates $\omega_{f}$, the primary surface noise can be modified (amplified or damped) by signals $s_{t}(\omega_{f})$ occurring at these points. Treating standardised signals $s_{t}^{*}(\omega_{f})=s_{t}(\omega_{f})/\sigma_{s}$ as noise-modifying functions, secondary noise $\vartheta_{t}$ at point $P_{f}$ is presented as

(2) $$\vartheta_{t}(\omega_{f})=e_{t,f}s_{t}^{*}(\omega_{f})$$

($\sigma_{s}$—signal’s standard deviation identical for each t∈T). Equation (2) results in the fact that secondary noise does not exist in two cases: (1) no object vibrations, hence no primary noise; and (2) there are vibrations generating noise, but their secondary version decays in the absence of signals.

Assume now that the secondary noise generated from other points on the surface reaches each specific point of surface π. At point $P_{i}\in \pi$, the total (resultant) noise presented in the following way can be expected:

(3) $${\bar{\vartheta }}_{i,t}^{}=\sum_{f=1}^{\infty }\vartheta_{i,t}^{}(\omega_{f})=\sum_{f=1}^{\infty }e_{i,t,f}^{}s_{t}^{*}(\omega_{f})$$

where $\vartheta_{i,t}^{}(\omega_{f})=e_{i,t,f}^{}s_{t}^{*}(\omega_{f})$ is the secondary noise generated at point $P_{f}$ reaching point $P_{i}$. The resultant of secondary noise ${\bar{\vartheta }}_{i,t}^{}$ and the signal $s_{t}(\omega_{i})$ form the total fluctuations of the $P_{i}$ point’s height on this surface, that is,

(4) $$\xi_{t}(\omega_{i})=s_{t}(\omega_{i})+{\bar{\vartheta }}_{i,t}^{}$$

In practice, the surface vertical deformations are determined on the basis of changes in heights H(t) of CPs on the control levelling network (in this paper, it is assumed that this is the FCN). Points $P_{k}$ of this network create a set $P=\{P_{1},\ldots ,P_{r}\}$, whereas their positions are determined by coordinates $\omega_{k}=(x_{k},y_{k})$ included in this set $\Omega =\{\omega_{1},\ldots ,\omega_{r}\}$. When surface points π are limited to the set P, then in accordance with Equation (3), the resultant of secondary noise reaching point $P_{k}\in P$ (CP) can be presented as

(5) $${\bar{\vartheta }}_{k,t}=\sum_{f=1}^{r}e_{k,t,f}^{}s_{t}^{*}(\omega_{f})=e_{k,t}^{T}s_{t}^{*}$$

where $e_{k,t}={\left[e_{k,t,1},\cdots ,e_{k,t,r}\right]}^{T}$, $s_{t}^{*}={\left[s_{1,t}^{*},\cdots ,s_{r,t}^{*}\right]}^{T}$, $s_{f,t}^{*}=s_{t}^{*}(\omega_{f})=s_{t}(\omega_{f})/\sigma_{s}$, f=1,…,r.

In deformation analysis, an interesting issue along with displacement and signals at CPs is also the prediction of displacements and signals at other non-measured points on the surface π. These points (relative to FCN) are referred to as ECPs. These points and their coordinates create the sets $P^{\prime }=\{P_{1}^{\prime },\ldots ,P_{z}^{\prime }\}$ and $\Omega^{\prime }=\{\omega_{1}^{\prime },\ldots ,\omega_{z}^{\prime }\}$, respectively. Moreover, assume that $s_{t}^{*}(\omega_{f}^{\prime })=s_{t}(\omega_{f}^{\prime })/\sigma_{s}$ is a standardised signal at point $P_{f}^{\prime }\in P^{\prime }$. Taking into account the ECPs, it leads to the resultant of secondary noise reaching the measured point $P_{k}\in P$ in the following, extended form

(6) $${\bar{\vartheta }}_{k,t}=\sum_{f=1}^{r}e_{k,t,f}s_{t}^{*}(\omega_{f})+\sum_{g=1}^{z}{e^{\prime }}_{k,t,g}s_{t}^{*}({\omega^{\prime }}_{f})=e_{k,t}^{T}s_{t}^{*}+{\left({e^{\prime }}_{k,t}^{}\right)}^{T}{\left({s^{\prime }}_{t}\right)}^{*}$$

where ${e^{\prime }}_{k,t}={\left[{e^{\prime }}_{k,t,1},\cdots ,{e^{\prime }}_{k,t,z}\right]}^{T}$, ${\left({s^{\prime }}_{t}\right)}^{*}={\left[{\left({s^{\prime }}_{1,t}\right)}^{*},\cdots ,{\left({s^{\prime }}_{z,t}\right)}^{*}\right]}^{T}$, ${\left({s^{\prime }}_{g,t}\right)}^{*}=s_{t}^{*}({\omega^{\prime }}_{g})=s_{t}({\omega^{\prime }}_{g})/\sigma_{s}$, g=1,…,z On the basis of Equations (4) and (6), total fluctuation of the heights of point $P_{k}\in P$ can be presented as

(7) $$\xi_{t}(\omega_{k})=s_{t}(\omega_{k})+{\bar{\vartheta }}_{k,t}=s_{t}(\omega_{k})+e_{k,t}^{T}s_{t}^{*}+{\left({e^{\prime }}_{k,t}^{}\right)}^{T}{\left({s^{\prime }}_{t}\right)}^{*}$$

For the signals $s_{t}(\omega_{f})$, $\omega_{f}\in \Omega \cup \Omega^{\prime }$, it is assumed that they form a stationary and isotropic process. In this case, the identical following covariance function for each t∈T can be assigned to these magnitudes [52]

(8) $$C(\omega_{i},\omega_{j})=C(\omega_{i},\omega_{i}+\Delta \omega )=C(\Delta \omega )=C(d_{i,j})=\sigma_{s}^{2}\rho (d_{i,j})$$

where $\omega_{i},\omega_{j}\in \Omega \cup \Omega^{\prime }$, $\Delta \omega =\omega_{j}-\omega_{i}$, and $d_{i,j}={\left\|\Delta \omega \right\|}^{1/2}$ are distance between any random pair of points belonging to the set $P\cup P^{\prime }$ ($\rho (d_{i,j})$—correlation function). The following values can be obtained using Function (8): covariances $\mathrm{cov}(s_{k},s_{l})=C(d_{k,l})$ of signals $s_{k}$ and $s_{l}$ at points $P_{k},P_{l}\in P$ (CPs), covariances $\mathrm{cov}({s^{\prime }}_{q},{s^{\prime }}_{p})=C(d_{q,p})$ of signals ${s^{\prime }}_{q}$ and ${s^{\prime }}_{p}$ at points $P_{q}^{\prime },P_{p}^{\prime }\in P^{\prime }$ (ECPs), and covariances $\mathrm{cov}({s^{\prime }}_{q},s_{k})=C(d_{q,k})$ among signals at points $P_{q}^{\prime }\in P^{\prime }$ and signals at points $P_{k}\in P$.

### 2.2. Heights and Displacements Models

Considering the total surface fluctuations, the height of point $P_{k}\in P$ lying on this surface is presented in the following form (based on Equations (1) and (7))

(9) $$ℋ(t,\omega_{k})=H_{k}(t)+\xi_{t}(\omega_{k})=H_{k}(t)+s_{t}(\omega_{k})+e_{k,t}^{T}s_{t}^{*}+{\left({e^{\prime }}_{k,t}^{}\right)}^{T}{\left({s^{\prime }}_{t}\right)}^{*}=H_{k,t}^{}+s_{k,t}^{}+e_{k,t}^{T}s_{t}^{*}+{\left({e^{\prime }}_{k,t}^{}\right)}^{T}{\left({s^{\prime }}_{t}\right)}^{*}$$

where $H_{k,t}=H_{k}(t)$ and $s_{k,t}=s_{t}(\omega_{k})$. After introducing a surface noise vector ${\overset{⌢}{e}}_{k,t}={\left[e_{k,t}^{T},{e^{\prime }}_{k,t}^{T}\right]}^{T}$, joint for CPs and ECPs, and their joint vector of standardised signals ${\overset{⌢}{s}}_{t}^{*}={\left[{\left(s_{t}^{*}\right)}^{T},{\left({\left({s^{\prime }}_{t}\right)}^{*}\right)}^{T}\right]}^{T}$, Equation (9) can also be written as

(10) $$ℋ(t,\omega_{k})=H_{k,t}^{}+s_{k,t}^{}+{\overset{⌢}{e}}_{k,t}^{T}{\overset{⌢}{s}}_{t}^{*}$$

In case of signals’ absence at all CPs and ECPs, model (10) is reduced to its classic form $ℋ(t,\omega_{k})=H_{k,t}$. This means that, in this case, even if there are surface vibrations and there is random noise coming from these vibrations, they do not contribute to the height model for point $P_{k}$. Consequently, similarly to random measurement errors, surface noise will disturb the observation (these magnitudes cannot be separated without additional assumptions). Whereas when $\forall f:e_{k,t,f}=0,\;{e^{\prime }}_{k,t,f}=0$, then surface fluctuation is composed only of signals, this means that $\xi_{t}(\omega_{k})=s_{t}(\omega_{k})$ takes place at point $P_{k}\in P$ and

(11) $$ℋ(t,\omega_{k})=H_{k,t}^{}+s_{k,t}^{}$$

In classic levelling networks forming the FCN, heights $H_{k,t}$ are not directly measured. They are treated as unknown parameters in functional observations models (the observations of heights differences between pairs of points). In this case, separating the signals from non-random parameters is a problem. Although it is possible to conduct the estimation of random parameters $ℋ(t,\omega_{k})=H_{k,t}^{}+s_{k,t}^{}=H_{k,t}^{rand}$ (e.g., applying the Bayes method, such as in [53,54]), the determination of the height estimator $H_{k,t}$ and the estimation of the signal $s_{k,t}$ related to this height remain an unsolved problem. In the proposed model (10), there is not only a signal at point $P_{k}$, but there are also signals at other CPs (and in ECPs) that, together with surface noise, form the resultant of secondary noise. Therefore, the direct connection of deterministic parameters and random signals is violated here. This creates an opportunity for separating them in the estimation process. Model (10) can be also applied in estimating the surfaces noise contained in vectors ${\overset{⌢}{e}}_{k,t}$.

Assume now that

(12) $$C_{\overset{⌢}{s}}=\left[\begin{array}{cc} C_{s} & C_{ss^{\prime }}\\ C_{s^{\prime }s} & C_{s^{\prime }} \end{array}\right]$$

is a covariance matrix of the joint signal vector ${\overset{⌢}{s}}_{t}={\left[s_{t}^{T},{\left({s^{\prime }}_{t}\right)}^{T}\right]}^{T}$ determined on the basis of the covariance function under Equation (8), where ${\left(C_{s}\right)}_{k,l}=\mathrm{cov}(s_{k},s_{l})$, ${\left(C_{s^{\prime }}\right)}_{q,p}=\mathrm{cov}({s^{\prime }}_{q},{s^{\prime }}_{p})$, ${\left(C_{s^{\prime }s}\right)}_{q,k}=\mathrm{cov}({s^{\prime }}_{q},s_{k})$, and $C_{s,s^{\prime }}=C_{s^{\prime },s}^{T}$ (${\left(C\right)}_{i,j}$—*i*, *j*th element of the matrix **C**). If the covariance matrix $C_{\overset{⌢}{s}}$ is positively defined, then there is a lower triangular matrix **R** for which $C_{\overset{⌢}{s}}=RR^{T}$ (Cholesky decomposition, for example, [54]). The vector created on its basis

(13) $${\overset{⌢}{s}}_{t}^{*}=R_{}^{-1}{\overset{⌢}{s}}_{t}$$

has a covariance matrix $C_{\overset{⌢}{s}*}=R_{}^{-1}C_{\overset{⌢}{s}}{\left(R_{}^{-1}\right)}^{T}=I_{r+z}$ identical for each t∈T ($I_{r+z}$—unit matrix of r+z). After Equation (13) is introduced into Equation (10), the result is

(14) $$ℋ(t,\omega_{k})=H_{k,t}^{}+s_{k,t}^{}+{\overset{⌢}{e}}_{k,t}^{T}R_{}^{-1}{\overset{⌢}{s}}_{t}$$

The levelling geodetic networks CPs’ displacements are calculated on the basis of the observations of the heights’ differences between these points, conducted during a few measurement epochs. Assume that the height difference $\Delta {ℋ}_{\left(k,l\right)}=ℋ(t,\omega_{l})-ℋ(t,\omega_{k})$ between points $P_{k},P_{l}\in P$ is the object of measurement in epochs $t_{\alpha },t_{\beta }\in T$. On the basis of Equation (14), the heights of these points in epoch $t_{\alpha }$ can be written as

(15) $$ℋ(t_{\alpha },\omega_{k})=H_{k,\alpha }^{}+s_{k,\alpha }^{}+{\overset{⌢}{e}}_{k,\alpha }^{T}R_{}^{-1}{\overset{⌢}{s}}_{\alpha },\;ℋ(t_{\alpha },\omega_{l})=H_{l,\alpha }^{}+s_{l,\alpha }^{}+{\overset{⌢}{e}}_{l,\alpha }^{T}R_{}^{-1}{\overset{⌢}{s}}_{\alpha }$$

In this case, the heights’ differences observation model $\Delta {ℋ}_{\left(k,l\right)}$ is formed as

(16) $$\begin{array}{cc} h_{i,\alpha }= & h_{(k,l),\alpha }=\Delta {ℋ}_{(k,l)\alpha }+v_{i,\alpha }=ℋ(t_{\alpha },\omega_{l})-ℋ(t_{\alpha },\omega_{k})+v_{i,\alpha }\\ = & H_{l,\alpha }-H_{k,\alpha }+s_{l,\alpha }-s_{k,\alpha }+{\overset{⌢}{e}}_{l,\alpha }^{T}R_{}^{-1}{\overset{⌢}{s}}_{\alpha }-{\overset{⌢}{e}}_{k,\alpha }^{T}R_{}^{-1}{\overset{⌢}{s}}_{\alpha }+v_{i,\alpha }\\ = & H_{l,\alpha }-H_{k,\alpha }+s_{l,\alpha }-s_{k,\alpha }+{\overset{⌢}{\varepsilon }}_{i,\alpha }^{T}R_{}^{-1}{\overset{⌢}{s}}_{\alpha }+v_{i,\alpha } \end{array}$$

where ${\overset{⌢}{\varepsilon }}_{i,\alpha }={\overset{⌢}{e}}_{l,\alpha }-{\overset{⌢}{e}}_{k,\alpha }$, i=(k,l). The random error of observation $h_{i,\alpha }$ is marked with $v_{i,\alpha }$ (with expected value $E(v_{i,\alpha })=0$ and variance $\sigma_{v}^{2}$). The elements of vector ${\overset{⌢}{\varepsilon }}_{i,\alpha }$ are differential noise with identical variances $\sigma_{E}^{2}=2\sigma_{e}^{2}$. The magnitudes of $H_{k,\alpha }$ and $H_{l,\alpha }$, k,l=1,…,r are the parameters of the functional model (16), determined by estimation. For total FCN, these parameters form vector $X_{\alpha }={\left[H_{1,\alpha },\cdots ,H_{r,\alpha }\right]}^{T}$. After using this vector, the model of *i*th observation can be presented as

(17) $$h_{i,\alpha }=a_{i}X_{\alpha }+a_{i}s_{\alpha }+{\overset{⌢}{\varepsilon }}_{i,\alpha }^{T}R_{}^{-1}{\overset{⌢}{s}}_{\alpha }+v_{i,\alpha }$$

where $a_{i}=[0,\cdots ,0,-1_{k},0,\cdots ,0,1_{l},0,\cdots ,0]$. When surface vibrations are absent, then ${\overset{⌢}{\varepsilon }}_{i,\alpha }=0$, hence

(18) $$h_{i,\alpha }=a_{i}X_{\alpha }+a_{i}s_{\alpha }+v_{i,\alpha }=a_{i}(X_{\alpha }+s_{\alpha })+v_{i,\alpha }=a_{i}X_{\alpha }^{rand}+v_{i,\alpha }$$

where $X_{\alpha }^{rand}=X_{\alpha }+s_{\alpha }$. In this specific case, the random signals are combined with parameters, which consequently leads to a random parameter $X_{\alpha }^{rand}$ with a covariance matrix of $C_{X_{\alpha }^{rand}}=C_{s}$.

Until this point, the signals in ECPs were treated only as the components of secondary noise reaching individual points within the FCN. Assume that these magnitudes, that is, signals at points $P_{1}^{\prime },\ldots ,P_{z}^{\prime }\in P^{\prime }$, will also be determined by estimation process. Then, according to the principles of collocation [32,33,34], model (17) can be supplemented to the following form

(19) $$\begin{array}{cc} h_{i,\alpha }=a_{i}X_{\alpha }+ & a_{i}s_{\alpha }+{\overset{⌢}{\varepsilon }}_{i,\alpha }^{T}R_{}^{-1}{\overset{⌢}{s}}_{\alpha }+0\cdot s_{\alpha }({\omega^{\prime }}_{1})+\cdots +0\cdot s_{\alpha }({\omega^{\prime }}_{z})+v_{i,\alpha }\\ = & a_{i}X_{\alpha }+a_{i}s_{\alpha }+{\overset{⌢}{\varepsilon }}_{i,\alpha }^{T}R_{}^{-1}{\overset{⌢}{s}}_{\alpha }+0_{z}^{T}{s^{\prime }}_{\alpha }+v_{i,\alpha } \end{array}$$

where $0_{z}={\left[0_{1},\cdots ,0_{z}\right]}^{T}$ is the zero vector (the zero component $0_{z}^{T}{s^{\prime }}_{\alpha }$ added to model (17) does not change the primary sense of this model). After further transformation of Equation (19), the formula is as follows

(20) $$\begin{array}{cc} h_{i,\alpha }=a_{i}X_{\alpha }+ & a_{i}s_{\alpha }+0_{z}^{T}{s^{\prime }}_{\alpha }+{\overset{⌢}{\varepsilon }}_{i,\alpha }^{T}R_{}^{-1}{\overset{⌢}{s}}_{\alpha }+v_{i,\alpha }=a_{i}X_{\alpha }+[a_{i},0_{z}^{T}]\left[\begin{array}{c} s_{\alpha }\\ {s^{\prime }}_{\alpha } \end{array}\right]+{\overset{⌢}{\varepsilon }}_{i,\alpha }^{T}R_{}^{-1}{\overset{⌢}{s}}_{\alpha }+v_{i,\alpha }\\ = & a_{i}X_{\alpha }+b_{i}{\overset{⌢}{s}}_{\alpha }+{\overset{⌢}{\varepsilon }}_{i,\alpha }^{T}R_{}^{-1}{\overset{⌢}{s}}_{\alpha }+v_{i,\alpha }=a_{i}X_{\alpha }+(b_{i}+{\overset{⌢}{\varepsilon }}_{i,\alpha }^{T}R_{}^{-1}){\overset{⌢}{s}}_{\alpha }+v_{i,\alpha } \end{array}$$

where $b_{i}=[a_{i},0_{z}^{T}]$. For total FCN, that is, including all observations $h_{i,\alpha }$, i=(k,l)=1,…,n, based on Equation (20), the following model is obtained

(21) $$y_{\alpha }=AX_{\alpha }+(B+{\overset{⌢}{E}}_{\alpha }R_{}^{-1}){\overset{⌢}{s}}_{\alpha }+v_{\alpha }=AX_{\alpha }+(B+{\overset{⌢}{E}}_{\alpha }){\overset{⌢}{s}}_{\alpha }+v_{\alpha }$$

where $y_{\alpha }={\left[h_{1,\alpha },\cdots ,h_{n,\alpha }\right]}^{T}$ is the observations vector and $v_{\alpha }={\left[v_{1,\alpha },\cdots ,v_{n,\alpha }\right]}^{T}$ is the vector of random observation errors with a covariance matrix of $C_{v}=\sigma_{v}^{2}I_{n}$. Matrix $A={\left[a_{1}^{T},\cdots ,a_{n}^{T}\right]}^{T}$ is a classic coefficient matrix in functional models of levelling networks. What is notable, in a levelling FCN, is that the **A** matrix is the matrix of vertically incomplete rank, that is, with rank(A)=u=r−d=r−1 (d=1—deficient rank). Furthermore, $B=[A,0_{n,z}]$ ($0_{n,z}$—zero matrix of n×z dimensions) and ${\overset{⌢}{E}}_{\alpha }={\left[{\overset{⌢}{\varepsilon }}_{1,\alpha }^{},\cdots ,{\overset{⌢}{\varepsilon }}_{n,\alpha }^{}\right]}^{T}=[E_{\alpha },{E^{\prime }}_{\alpha }]$. The ${\overset{⌢}{E}}_{\alpha }$ matrix is a matrix of random noise as a result of surface vibrations, whereas $E_{\alpha }={\left[\varepsilon_{1,\alpha }^{},\cdots ,\varepsilon_{n,\alpha }^{}\right]}^{T}$ applies to noise at CPs and ${E^{\prime }}_{\alpha }={\left[{\varepsilon^{\prime }}_{1,\alpha },\cdots ,{\varepsilon^{\prime }}_{n,\alpha }\right]}^{T}$ is the noise matrix at ECPs. All elements of these matrices have common variance $\sigma_{E}^{2}=2\sigma_{e}^{2}$. Matrix ${\overset{⌢}{E}}_{\alpha }={\overset{⌢}{E}}_{\alpha }R_{}^{-1}=[E_{\alpha },{E^{\prime }}_{\alpha }]$, where $E_{\alpha }=E_{\alpha }R_{}^{-1}$, ${E^{\prime }}_{\alpha }={E^{\prime }}_{\alpha }R_{}^{-1}$, is a matrix that disturbs the corresponding blocks of **B** matrix ($E_{\alpha }$ disturbs the **A** matrix, whereas ${E^{\prime }}_{\alpha }$ disturbs the zero matrix $0_{n,z}$).

Similarly to above, the determination of the model of observation vector $y_{\beta }$ in epoch $t_{\beta }$, that is, based on Equation (21), gives

(22) $$y_{\beta }=AX_{\beta }+(B+{\overset{⌢}{E}}_{\beta }){\overset{⌢}{s}}_{\beta }+v_{\beta }$$

(index α is substituted here with index β indicating the current measurement epoch).

Assume that the network points’ displacements are determined on the basis of difference $\delta_{y}=y_{\beta }-y_{\alpha }$ of observation vectors in epochs $t_{\alpha }$ and $t_{\beta }$. Using Equations (21) and (22), the following model of this difference is obtained

(23) $$\delta_{y}=Au+(B+{\overset{⌢}{E}}_{\beta })s_{\beta }-(B+{\overset{⌢}{E}}_{\alpha })s_{\alpha }+\upsilon$$

where $u=X_{\beta }-X_{\alpha }$ is the CPs’ vertical displacement in these epochs. The magnitude u will be also treated as the deterministic parameter shift (e.g., [14]). Vector $\upsilon =v_{\beta }-v_{\alpha }$ is the vector of the differences of the observations errors with a covariance matrix of $C_{\upsilon }=2\sigma_{v}^{2}I_{n}$.

In model (23), it is assumed that disturbances of the B matrix differ in epochs $t_{\alpha }$ and $t_{\beta }$. In practice, however, it is possible to adopt the assumption that object vibrations in both of these epochs are similar. In this case, it can be expected that primary noise $e_{t}$ will have similar values coming from these vibrations. It should be noted that, even with this simplification, fluctuations of heights of the points in epochs $t_{\alpha }$ and $t_{\beta }$ can differ from one another. Signals $s_{t}({\overset{⌢}{\omega }}_{f})$ modifying primary noise (see Equation (2)) remain dependent on time. The assumptions made are not general in nature (i.e., they do not correspond to each practical situation), but they can significantly simplify model (23). Without this simplification, it may be difficult to estimate surface noise. Applying equation $\overset{⌢}{E}={\overset{⌢}{E}}_{\alpha }={\overset{⌢}{E}}_{\beta }$, and thus adopting identical disturbs of B matrix in both measurement epochs, that is, $\overset{⌢}{E}={\overset{⌢}{E}}_{\alpha }={\overset{⌢}{E}}_{\beta }$, the model of the vector of the observations’ differences of heights’ differences in epochs $t_{\alpha }$ and $t_{\beta }$ can be expressed as

(24) $$\delta_{y}=Au+(B+E)({\overset{⌢}{s}}_{\beta }-{\overset{⌢}{s}}_{\alpha })+\upsilon =Au+(B+\overset{⌢}{E})\overset{⌢}{\eta }+\upsilon$$

Vector $\overset{⌢}{\eta }={\overset{⌢}{s}}_{\beta }-{\overset{⌢}{s}}_{\alpha }={\left[\eta^{T},{\left(\eta^{\prime }\right)}^{T}\right]}^{T}$, where $\eta =s_{\beta }-s_{\alpha }$, $\eta^{\prime }={s^{\prime }}_{\beta }-{s^{\prime }}_{\alpha }$ is a vector of differential signals with zero expected values and covariance matrix of $C_{\overset{⌢}{\eta }}=2C_{\overset{⌢}{s}}$. Its elements can be also treated as signals for points‘ displacements in epochs $t_{\alpha }$ and $t_{\beta }$. Vector η is thus the vector of CPs’ random displacement, which can be used to determine the total displacements $u_{\eta }=u+\eta$ of these points, in conjunction with deterministic displacements u. Meanwhile, the $\eta^{\prime }$ vector is the vector of predictions of random ECPs’ displacements. The deterministic displacements of these points are not parameters in model (24), hence they are not determined by estimation process. However, if it is assumed that ${u^{\prime }}_{ECP}$ is the vector of interpolated ECPs’ displacements (calculated on the basis of the displacements of CPs located in their proximity), then their total displacements can be expressed as ${u^{\prime }}_{\eta ,ECP}={u^{\prime }}_{ECP}+\eta^{\prime }$.

For the purpose of further optimisation process, the $\overset{⌢}{E}$ matrix can be conveniently substituted with a vector created from its subsequent columns, that is, with vector

(25) $$v_{\overset{⌢}{E}}=\mathrm{vec}(\overset{⌢}{E})={\left[\mathrm{vec}{\left(E\right)}^{T},\mathrm{vec}{\left(E^{\prime }\right)}^{T}\right]}^{T}$$

with a covariance matrix of

(26) $$C_{v_{\overset{⌢}{E}}}=\sigma_{E}^{2}I_{n\cdot (r+z)}=\sigma_{E}^{2}(I_{n+z}⊗I_{n})$$

(⊗ Kronecker product). Applying the following, general dependence (e.g., [49,51,55])

(27) $$\mathrm{vec}(ABC)=(C^{T}⊗A)\mathrm{vec}(B)$$

the expression can be converted to $\overset{⌢}{E}\overset{⌢}{\eta }=\overset{⌢}{E}R^{-1}\overset{⌢}{\eta }$ as

(28) $$\overset{⌢}{E}\overset{⌢}{\eta }=\overset{⌢}{E}R^{-1}\overset{⌢}{\eta }=I_{n}\overset{⌢}{E}(R^{-1}\overset{⌢}{\eta })=({\overset{⌢}{\eta }}^{T}R^{-1}⊗I_{n})v_{\overset{⌢}{E}}$$

Introducing the above transformation into model (24), the result is as follows

(29) $$\delta_{y}=Au+(B+\overset{⌢}{E})\overset{⌢}{\eta }+\upsilon =Au+B\overset{⌢}{\eta }+({\overset{⌢}{\eta }}^{T}R^{-1}⊗I_{n})v_{\overset{⌢}{E}}+\upsilon$$

## 3. Optimisation Problem and Its Solution

The unknown elements of model (29) can be determined according to the principles of the TLS method (e.g., [49]). The following optimisation criterion is adopted

(30) $$\varphi (\upsilon ,v_{\overset{⌢}{E}},\overset{⌢}{\eta },u,\lambda )=\upsilon^{T}C_{\upsilon }^{-1}\upsilon +v_{\overset{⌢}{E}}^{T}C_{v_{\overset{⌢}{E}}}^{-1}v_{\overset{⌢}{E}}+{\overset{⌢}{\eta }}^{T}C_{\overset{⌢}{\eta }}^{-1}\overset{⌢}{\eta }-2\lambda^{T}\left(W(\upsilon ,v_{\overset{⌢}{E}},\overset{⌢}{\eta },u)\right)=\min$$

where

(31) $$W(\upsilon ,v_{\overset{⌢}{E}},\overset{⌢}{\eta },u)=Au+(B+\overset{⌢}{E})\overset{⌢}{\eta }+\upsilon -\delta_{y}=Au+B\overset{⌢}{\eta }+({\overset{⌢}{\eta }}^{T}R^{-1}⊗I_{n})v_{\overset{⌢}{E}}+\upsilon -\delta_{y}=0$$

is the equation that links the determined magnitudes (λ—vector of Lagrange multipliers).

The optimisation problem $\varphi (\upsilon ,v_{\overset{⌢}{E}},\overset{⌢}{\eta },u,\lambda )=\min$ is solved by such magnitudes as $\hat{\upsilon },{\hat{v}}_{\overset{⌢}{E}},\hat{\overset{⌢}{\eta }},\hat{u}$, for which the following Euler–Lagrange conditions are fulfilled (e.g., [48,49,50])

(32) $${\frac{\partial \varphi }{\partial \hat{\upsilon }}|}_{\hat{\upsilon },{\hat{v}}_{\overset{⌢}{E}},\hat{\overset{⌢}{\eta }},\hat{u},\hat{\lambda }}=2C_{\upsilon }^{-1}\hat{\upsilon }-2\hat{\lambda }=0$$

(33) $${\frac{\partial \varphi }{\partial {\hat{v}}_{\overset{⌢}{E}}}|}_{\hat{\upsilon },{\hat{v}}_{\overset{⌢}{E}},\hat{\overset{⌢}{\eta }},\hat{u},\hat{\lambda }}=2C_{v_{\overset{⌢}{E}}}^{-1}{\hat{v}}_{\overset{⌢}{E}}-2(R^{-1}\hat{\overset{⌢}{\eta }}⊗I_{n})\hat{\lambda }=0$$

(34) $${\frac{\partial \varphi }{\partial \overset{⌢}{\eta }}|}_{\hat{\upsilon },{\hat{v}}_{\overset{⌢}{E}},\hat{\overset{⌢}{\eta }},\hat{u},\hat{\lambda }}=2C_{\overset{⌢}{\eta }}^{-1}\hat{\overset{⌢}{\eta }}-2{\left(B+\hat{\overset{⌢}{E}}\right)}^{T}\hat{\lambda }=0$$

(35) $${\frac{\partial \varphi }{\partial u}|}_{\hat{\upsilon },{\hat{v}}_{\overset{⌢}{E}},\hat{\overset{⌢}{\eta }},\hat{u},\hat{\lambda }}=2A^{T}\hat{\lambda }=0$$

(36) $${\frac{\partial \varphi }{\partial \lambda }|}_{\hat{\upsilon },{\hat{v}}_{\overset{⌢}{E}},\hat{\overset{⌢}{\eta }},\hat{u},\hat{\lambda }}=A\hat{u}+(B+\hat{\overset{⌢}{E}})\hat{\overset{⌢}{\eta }}+\hat{\upsilon }-\delta_{y}=0$$

Furthermore, the necessary conditions used to positively define second derivatives must be fulfilled here

(37) $$\frac{\partial^{2}\varphi }{\partial \hat{\upsilon }\partial {\hat{\upsilon }}^{T}}=2C_{\upsilon }^{-1},\frac{\partial^{2}\varphi }{\partial v_{E}\partial v_{E}^{T}}=2C_{v_{E}}^{-1},\frac{\partial^{2}\varphi }{\partial \eta \partial \eta^{T}}=2C_{\eta }^{-1}$$

The following residual vector is a result of Equation (32) and concerns the vector of observation differences in epochs $t_{\alpha }$ and $t_{\beta }$

(38) $$\hat{\upsilon }=C_{\upsilon }\hat{\lambda }$$

Meanwhile, applying Equation (33) and taking into consideration the covariance matrix (26), the following equation will be obtained

(39) $${\hat{v}}_{\overset{⌢}{E}}=C_{v_{\overset{⌢}{E}}}(R^{-1}\hat{\overset{⌢}{\eta }}⊗I_{n})\hat{\lambda }=\sigma_{E}^{2}(I_{n+z}⊗I_{n})(R^{-1}\hat{\overset{⌢}{\eta }}⊗I_{n})\hat{\lambda }=\sigma_{E}^{2}(R^{-1}\hat{\overset{⌢}{\eta }}⊗I_{n})\hat{\lambda }$$

Because ${\hat{v}}_{\overset{⌢}{E}}=\mathrm{vec}(\hat{\overset{⌢}{E}})$ and $\hat{\lambda }=\mathrm{vec}(\hat{\lambda })$, and based on Equation (27), the following matrix is determined

(40) $$\hat{\overset{⌢}{E}}=\sigma_{E}^{2}\hat{\lambda }{\hat{\overset{⌢}{\eta }}}^{T}{\left(R^{-1}\right)}^{T}=[\hat{E},{\hat{E}}^{\prime }]$$

which constitutes the assessment of the noise matrix in CPs ($\hat{E}$) and in ECPs (${\hat{E}}^{\prime }$). Therefore, the matrix that disturbs corresponding matrix blocks $B=[A,0_{n,z}]$ is

(41) $$\hat{\overset{⌢}{E}}=\hat{\overset{⌢}{E}}R^{-1}=\sigma_{E}^{2}\hat{\lambda }{\hat{\overset{⌢}{\eta }}}^{T}{\left(R^{-1}\right)}^{T}R^{-1}=\sigma_{E}^{2}\hat{\lambda }{\hat{\overset{⌢}{\eta }}}^{T}C_{\overset{⌢}{s}}^{-1}=[\hat{E},{\hat{E}}^{\prime }]$$

Using Equation (35), the following vector can be determined

(42) $$\hat{\overset{⌢}{\eta }}=C_{\overset{⌢}{\eta }}{\left(B+\hat{\overset{⌢}{E}}\right)}^{T}\hat{\lambda }={\left[{\hat{\eta }}^{T},{\left({\hat{\eta }}^{\prime }\right)}^{T}\right]}^{T}$$

Its corresponding blocks are the estimations of CPs’ random displacements (vector $\hat{\eta }$) and prediction of ECPs’ random displacements (vector ${\hat{\eta }}^{\prime }$). Inserting Equations (38) and (42) into Equation (36), the result is

(43) $$(B+\hat{\overset{⌢}{E}})C_{\overset{⌢}{\eta }}{\left(B+\hat{\overset{⌢}{E}}\right)}^{T}\hat{\lambda }+C_{\upsilon }\hat{\lambda }+A\hat{u}-\delta_{y}=D\hat{\lambda }+A\hat{u}-\delta_{y}=0$$

where

(44) $$D=(B+\hat{\overset{⌢}{E}})C_{\overset{⌢}{\eta }}{\left(B+\hat{\overset{⌢}{E}}\right)}^{T}+C_{\upsilon }$$

If rank(D)=n, then the solution of Equation (43) is the following vector of Lagrange multipliers

(45) $$\hat{\lambda }=-D^{-1}(A\hat{u}-\delta_{y})$$

After inserting Equation (45) into Equation (35), the following equation is obtained

(46) $$A^{T}D^{-1}A\hat{u}-A^{T}D^{-1}\delta_{y}=0$$

which can be used to determine the estimator $\hat{u}$ of the vector of deterministic displacements u.

Because $\mathrm{rank}(A^{T}D^{-1}A)=\mathrm{rank}(A)=r-1$, then any of the g-inverse of matrix $A^{T}D^{-1}A$, should be used to solve Equation (46). In free networks adjustment, the following g-inverse of minimum norm, is particularly significant (e.g., [21,22,23,24,25,56])

(47) $${\left(A^{T}D^{-1}A\right)}^{+}=A^{T}D^{-1}A{\left(A^{T}D^{-1}A\cdot A^{T}D^{-1}A\right)}^{-}$$

The estimator $\hat{u}$ determined with the use of this inverse fulfils the optimisation criterion ${\left\|u\right\|}^{2}=u^{T}u=\min$ (other criteria are also used in similar problems, see, for example, [17]). Because the rank of **A** matrix is known, the method based on the presentation of this matrix in form of block $A=[A_{a},A_{b}]$ can be used to calculate ${\left(A^{T}D^{-1}A\right)}^{+}$. The $A_{a}$ matrix is a matrix with r−1 columns, for which $\mathrm{rank}(A_{a})=\mathrm{rank}(A)$, whereas $A_{b}$ is a single-column matrix (e.g., [14,25,57]). Then,

(48) $${\left(A^{T}D^{-1}A\right)}^{+}=\left[\begin{array}{cc} A_{a}^{T}D^{-1}A_{a}\cdot {\left(GG^{T}\right)}^{-1} & 0_{r-1,1}\\ A_{b}^{T}D^{-1}A_{a}\cdot {\left(GG^{T}\right)}^{-1} & 0 \end{array}\right]$$

where $G=\begin{array}{cc} {}[A_{a}^{T}D^{-1}A_{a} & A_{a}^{T}D^{-1}A_{b} \end{array}]$. After applying Equation (48), the solution of Equation (46) is the following estimator of CPs’ deterministic displacements

(49) $$\hat{u}={\left(A^{T}D^{-1}A\right)}^{+}A^{T}D^{-1}\delta_{y}=G^{T}{\left(GG^{T}\right)}^{-1}A_{a}^{T}D^{-1}\delta_{y}$$

The expressions obtained above indicate that the solution to the problem $\varphi (\upsilon ,v_{\overset{⌢}{E}},\overset{⌢}{\eta },u,\lambda )=\min$ is non-linear. It is required to use an iterative procedure with a properly selected starting step and a rational stopping criterion. Owing to Equations (40) and (42), the starting matrix of random noise $\overset{⌢}{E}$ plays a primary role in initiating iteration. Assume that ${\overset{⌢}{E}}^{\left(0\right)}=0$ is this matrix. Then, ${\overset{⌢}{E}}^{\left(0\right)}={\overset{⌢}{E}}^{\left(0\right)}R^{-1}=0$ and

(50) $${\overset{⌢}{\eta }}^{\left(0\right)}=C_{\overset{⌢}{\eta }}{\left(B+{\overset{⌢}{E}}^{\left(0\right)}\right)}^{T}\lambda =C_{\overset{⌢}{\eta }}B^{T}\lambda =C_{\overset{⌢}{\eta }}{\left[A,0_{n,z}^{}\right]}^{T}\lambda$$

Because $A^{T}\lambda =0$ (see Equation (35)) and $0_{n,z}^{T}\lambda =0$, then it is obtained that ${\overset{⌢}{\eta }}^{\left(0\right)}=0$ for matrix ${\overset{⌢}{E}}^{\left(0\right)}=0$. This means that, for this initial noise matrix, the iteration process will not start. A different, non-zero matrix ${\overset{⌢}{E}}^{\left(0\right)}$ should be thus adopted for the initiation of this process. On the other hand, there can be cases where there is no object’s noise, in that case, $\overset{⌢}{E}=0$. For this reason, the authors propose that the elements of the starting matrix ${\overset{⌢}{E}}^{\left(0\right)}$ should be small enough not to disturb the structure of observation (in the case of the absence of noise), and large enough to ensure that the iteration process is able to start (if noise exists). The authors propose that, according to the adopted nature of the noise matrix $\overset{⌢}{E}$, matrix ${\overset{⌢}{E}}^{\left(0\right)}$ should be simulated with the use of a generator of Gaussian’s random matrices with a standard deviation common to all of its elements $\sigma_{E^{\left(0\right)}}=c\sigma_{E}$. The c coefficient should be selected in such way that ${\overset{⌢}{E}}^{\left(0\right)}$ fulfils the aforementioned requirements (e.g., c=0.01).

For the adopted starting noise matrix and starting disturbance matrix ${\overset{⌢}{E}}^{\left(0\right)}={\overset{⌢}{E}}^{\left(0\right)}R_{}^{-1}$, the following iterative algorithm (i=0,1,2,…,m) is proposed:

(51) $$D^{\left(i+1\right)}=(B+{\overset{⌢}{E}}^{\left(i\right)})C_{\overset{⌢}{\eta }}{\left(B+{\overset{⌢}{E}}^{\left(i\right)}\right)}^{T}+C_{\upsilon }$$

(52) $$G^{\left(i+1\right)}=\begin{array}{cc} {}[A_{a}^{T}{\left(D^{\left(i+1\right)}\right)}^{-1}A_{a} & A_{a}^{T}{\left(D^{\left(i+1\right)}\right)}^{-1}A_{b} \end{array}]$$

(53) $$u^{\left(i+1\right)}={\left(G^{\left(i+1\right)}\right)}^{T}{\left(G^{\left(i+1\right)}G^{\left(i+1\right)}{}^{T}\right)}^{-1}A_{a}^{T}{\left(D^{\left(i+1\right)}\right)}^{-1}\delta_{y}$$

(54) $$\lambda^{\left(i+1\right)}=-{\left(D^{\left(i+1\right)}\right)}^{-1}(Au^{\left(i+1\right)}-\delta_{y})$$

(55) $$\upsilon^{\left(i+1\right)}=C_{\upsilon }\lambda^{\left(i+1\right)}$$

(56) $${\overset{⌢}{\eta }}^{\left(i+1\right)}=C_{\overset{⌢}{\eta }}{\left(B+{\overset{⌢}{E}}^{\left(i\right)}\right)}^{T}\lambda^{\left(i+1\right)}$$

(57) $${\overset{⌢}{E}}^{\left(i+1\right)}=\sigma_{E}^{2}\lambda^{\left(i+1\right)}{\left({\overset{⌢}{\eta }}^{\left(i+1\right)}\right)}^{T}$$

(58) $${\overset{⌢}{E}}^{i+1}={\overset{⌢}{E}}^{\left(i+1\right)}R^{-1}$$

Iteration ends for such *m* that $\left\|u^{\left(m\right)}-u^{\left(m-1\right)}\right\|<\xi_{u}$, $\left\|\lambda^{\left(m\right)}-\lambda^{\left(m-1\right)}\right\|<\xi_{\lambda }$, $\left\|{\overset{⌢}{\eta }}^{\left(m\right)}-{\overset{⌢}{\eta }}^{\left(m-1\right)}\right\|<\xi_{\eta }$ and $\left\|{\overset{⌢}{E}}^{\left(m\right)}-{\overset{⌢}{E}}^{\left(m-1\right)}\right\|<\xi_{E}$ (for given $\xi_{u}$, $\xi_{\lambda }$, $\xi_{\eta }$, $\xi_{E}$).

## 4. Numerical Tests

### 4.1. Simulated Levelling Network

**Figure 1:**
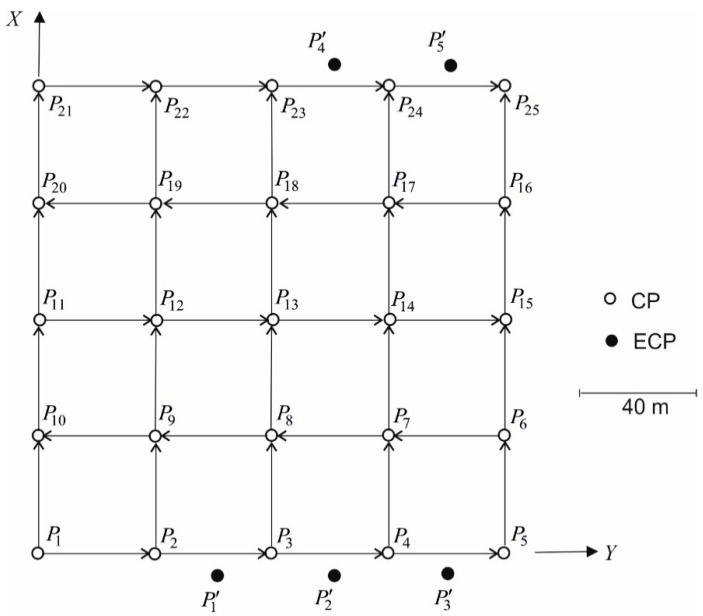
Simulated free control network. CP, controlled point; ECP, extended CP.

Basic numerical tests were carried out on the example of simulated levelling network (Figure 1) constituting the FCN. This network is composed of a set of $P=\{P_{1},\ldots ,P_{r}\}$, r=25, CPs and a set of $P_{\prime }=\{P_{1}^{\prime },\ldots ,P_{z}^{\prime }\}$, z=5, ECPs. The positions of these points are defined by their coordinates, $\omega_{k}=(x_{k},y_{k})\in \Omega$ and ${\omega^{\prime }}_{l}=(x_{l},y_{l})\in \Omega^{\prime }$ (for k=1,…,25 and l=26,…,30), respectively. It was assumed that the network is measured in two epochs, $t_{\alpha }$ and $t_{\beta }$. In each of these epochs, the observations of heights differences $h_{i}$, i=1,…,40 between CPs were simulated by adding the observations errors $v_{i}$ of normal distribution $N(0,\sigma_{v}^{2})$ to their theoretical values, where $\sigma_{v}$ is the assumed standard deviation of these errors. These errors were generated using a random number generator $\sigma_{v}randn(n,1)$, n=40 included in the MatLab package. Observation vectors $y_{\alpha }={\left[h_{1,\alpha },\cdots ,h_{40,\alpha }\right]}^{T}$ and $y_{\beta }={\left[h_{1,\beta },\cdots ,h_{40,\beta }\right]}^{T}$ were obtained this way and then used to calculate the vector of observations differences $\delta_{y}=y_{\beta }-y_{\alpha }$ in epochs $t_{\alpha }$ and $t_{\beta }$.

The simulation of differential signals in CPs and in ECPs, that is, the random displacements of these points, required the application of the following procedure:

1. Generation of mutually independent elements of vectors, ${\overset{⌢}{s}}_{t}^{*}={\left[{\left(s_{t}^{*}\right)}^{T},{\left({\left(s_{t}^{\prime }\right)}^{*}\right)}^{T}\right]}^{T}\sim N(0,I_{30})$, $t=t_{\alpha },t_{\beta }$ using generator randn(r+z,1).
2. Creation of a covariance matrix $C_{\overset{⌢}{s}}$ of signals vector ${\overset{⌢}{s}}_{t}={\left[s_{t}^{T},{\left({s^{\prime }}_{t}\right)}^{T}\right]}^{T}$ for the adopted covariance function $C(d_{i,j})=\sigma_{s}^{2}\rho (d_{i,j})$, i,j=1,…,30.
3. Calculation of signals vectors ${\overset{⌢}{s}}_{t}=R{\overset{⌢}{s}}_{t}^{*}\sim N(0,C_{\overset{⌢}{s}})$, $t=t_{\alpha },t_{\beta }$, based on the **R** matrix of $C(d_{i,j})=\sigma_{s}^{2}\rho (d_{i,j})=\sigma_{s}^{2}\exp(-kd_{i,j}^{2})$, $C_{\overset{⌢}{s}}=RR^{T}$ distribution and simulated vectors ${\overset{⌢}{s}}_{t}^{*}$.
4. Calculation of simulated random displacements vector of $\overset{⌢}{\eta }=({\overset{⌢}{s}}_{\beta }-{\overset{⌢}{s}}_{\alpha }\sim N(0,2C_{\overset{⌢}{s}})$.

Matrix of random noise resulting from surface vibrations, that is, the $\overset{⌢}{E}=[E,E^{\prime }]$ matrix was simulated using generator $\sigma_{E}randn(n,r+z)$. This matrix is the basis for the calculation of the $\overset{⌢}{E}=\overset{⌢}{E}R_{}^{-1}$ matrix that disturbs the B matrix in model (24).

The Gaussian’s covariance function of signals was adopted in the tests [41,42,54,58] in the following form

(59) $$C(d_{i,j})=\sigma_{s}^{2}\rho (d_{i,j})=\sigma_{s}^{2}\exp(-kd_{i,j}^{2})$$

The k coefficient in the correlation function $\rho (d_{i,j})=\exp(-kd_{i,j}^{2})$ was determined in such way that, for the most distant points of network $d_{\max}=230\;m$, the correlation coefficient between the signals at these points has the assumed minimum value $\rho_{\min}$. The value of this coefficient must be selected in such way that the covariance matrix $C_{\overset{⌢}{s}}$ is positively defined (too high a value of $\rho_{\min}$ leads to the negative definitiveness). For instance, $k=-\ln(\rho_{\min})/d_{\max}^{2}=8.7\cdot 10^{-5}$ is obtained for $\rho_{\min}=0.01$. There are also functions that belong to other classes of theoretical covariance functions and can be applied in practice. The chosen function should, however, correspond to the unique engineering object’s specification as well as its monitoring programme and technical conditions.

The influence of not recognising random displacements and surface noise on estimators of the **u** parameter in functional observation models was verified. For this reason, an analysis applying the Crude version of the Monte Carlo method was carried out. For N=5000 of independent simulations of vectors $v_{\alpha ,sim}$,$v_{\beta ,sim}$, ${\overset{⌢}{s}}_{\alpha ,sim}$, and ${\overset{⌢}{s}}_{\beta ,sim}$ and matrices ${\overset{⌢}{E}}_{sim}$, the theoretical vector of differential observations ${\bar{\delta }}_{y}=A\bar{u}$ was loaded with a random vector $\xi_{sim}^{i}=(B+{\overset{⌢}{E}}_{}^{i}){\overset{⌢}{\eta }}_{sim}^{i}+\upsilon_{sim}^{i}$, where ${\overset{⌢}{E}}^{i}={\overset{⌢}{E}}_{sim}^{i}R^{-1}$, $\eta_{sim}^{i}=s_{\beta ,sim}^{i}-s_{\alpha ,sim}^{i}$, and $\upsilon_{sim}^{i}=v_{\beta ,sim}^{i}-v_{\alpha ,sim}^{i}$ (for each i=1,…,N). The $\bar{u}$ vector is the adopted theoretical displacement of CPs. LS-estimators ${\hat{u}}^{i}$ of u parameter were determined on the basis of the model $\delta_{y}=Au+\upsilon$, for the simulated observations $\delta_{y,sim}^{i}=A\bar{u}+\xi_{sim}^{i}$. It should be noted that the model adopted in this paper does not take into account the signals and noise, although observations tend to be burdened by them. For comparison, the ${\hat{u}}_{p}^{i}$ LS-estimators determined on the basis of observations burdened only with the vector of differential observation errors $\upsilon_{sim}^{i}$ (without signals and noise) were also calculated. The estimators ${\hat{u}}^{i}$ and ${\hat{u}}_{p}^{i}$, i=1,…,N, were the basis for calculating the magnitudes of

(60) $$J_{\hat{u}}^{MC}=\sum_{i=1}^{N}{\left({\left\|\bar{u}-{\hat{u}}^{i}\right\|}^{2}/r\right)}^{1/2}/N,J_{{\hat{u}}_{p}}^{MC}=\sum_{i=1}^{N}{\left({\left\|\bar{u}-{\hat{u}}_{p}^{i}\right\|}^{2}/r\right)}^{1/2}/N$$

which constitute the Monte Carlo assessment of the following comparative parameters

(61) $$J_{\hat{u}}={\left({\left\|\bar{u}-{\hat{u}}^{i}\right\|}^{2}/r\right)}^{1/2},J_{{\hat{u}}_{p}}^{}={\left({\left\|\bar{u}-{\hat{u}}_{p}^{i}\right\|}^{2}/r\right)}^{1/2}$$

Calculations were carried out for three versions of the vector $\bar{u}$

$$\bar{u}={\left[{\bar{u}}_{1}^{},\;{\bar{u}}_{2}^{},\;{\bar{u}}_{3}^{},\;\cdots ,\;{\bar{u}}_{25}^{}\right]}^{T}=\{\begin{array}{l} {\left[0,\;0,\;0,\;\cdots \;,\;0\right]}^{T}{}_{\mathrm{mm}}\\ {\left[-5,\;0,\;0,\;\cdots \;,\;0\right]}^{T}{}_{\mathrm{mm}}\\ {\left[-10,\;0,\;0,\;\cdots \;,\;0\right]}^{T}{}_{\mathrm{mm}} \end{array}$$

**Figure 2:**
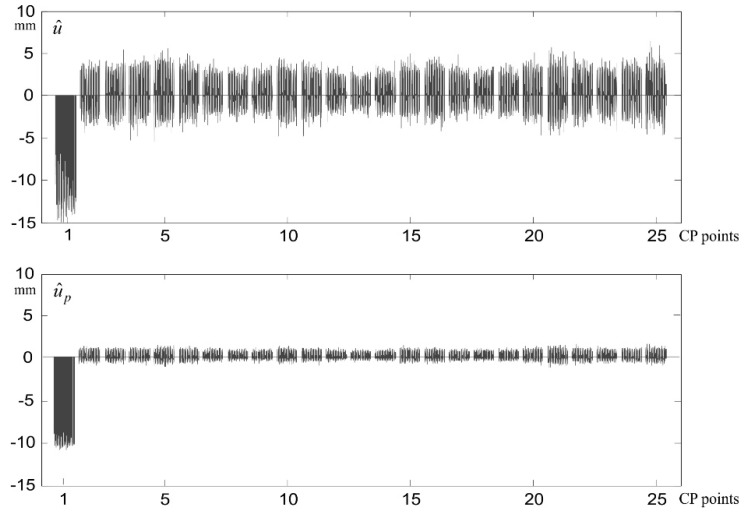
Estimators ${\hat{u}}_{k}^{i}$ and ${\hat{u}}_{p,k}^{i}$, k=1,…,25 of deterministic displacements of CPs, obtained in the sequence i=1,…,5000 of simulations (for ${\bar{u}}_{1}=10\;\mathrm{mm}$, $\sigma_{v}=0.3\;\mathrm{mm}$, $\sigma_{s}=1.0\;\mathrm{mm}$, and $\sigma_{E}=0.1\;\mathrm{mm}$).

Estimators ${\hat{u}}^{i}={\left[{\hat{u}}_{1}^{i},\;\cdots ,\;{\hat{u}}_{25}^{i}\right]}^{T}$ and ${\hat{u}}_{p}^{i}={\left[{\hat{u}}_{p,1}^{i},\;\cdots ,\;{\hat{u}}_{p,25}^{i}\right]}^{T}$ were determined for different values of signal’s standard deviations (the same for each measurement epoch): $\sigma_{s}=0\;\mathrm{mm}$ (no signals), $\sigma_{s}=0.5\;\mathrm{mm}$, and $\sigma_{s}=1.0\;\mathrm{mm}$, and for different values of surface noise’s standard deviations: $\sigma_{E}=0\;\mathrm{mm}$ (no noise), $\sigma_{E}=0.1\;\mathrm{mm}$, $\sigma_{E}=0.2\;\mathrm{mm}$, and $\sigma_{E}=0.3\;\mathrm{mm}$. In each case, an identical standard deviation of observations errors $\sigma_{v}=0.3\;\mathrm{mm}$ was adopted. The values of $J_{\hat{u}}^{MC}$ and $J_{{\hat{u}}_{p}}^{MC}$ obtained using the Monte Carlo (MC) method are presented in Table 1 (all values are expressed in mm). Figure 2 presents the courses of estimators ${\hat{u}}_{k}^{i}$ and ${\hat{u}}_{p,k}^{i}$ obtained in the sequence i=1,…,N of simulations (for variants ${\bar{u}}_{1}=10\;\mathrm{mm}$, $\sigma_{v}=0.3\;\mathrm{mm}$, $\sigma_{s}=1.0\;\mathrm{mm}$, and $\sigma_{E}=0.1\;\mathrm{mm}$).

The obtained results indicate that the differential signals (random displacement) omitted in the functional model can significantly affect the displacements values of the control network points that are determined with the use of this model. According to the theoretical assumptions, this influence grows together with the increase of noise values produced by surface vibrations. For instance, for noise with standard deviations $\sigma_{E}=0.1\;\mathrm{mm}$ and $\sigma_{E}=0.3\;\mathrm{mm}$, the difference between comparative parameters $J_{\hat{u}}^{MC}$ and $J_{{\hat{u}}_{p}}^{MC}$ will be 0.60 mm and 1.18 mm (for the variant, where ${\bar{u}}_{1}=10\;\mathrm{mm}$ and $\sigma_{s}=1.0\;\mathrm{mm}$), respectively. What is noteworthy is that the influence of signals is present also when there is no noise. In the discussed variant, $J_{\hat{u}}^{MC}-J_{{\hat{u}}_{p}}^{MC}=0.49$ was obtained for $\sigma_{E}=0\;\mathrm{mm}$. However, in this and similar examples, there is an issue with separating random signals from deterministic displacements (which was discussed in the theoretical part of the paper).

Both differential signals and surface noise are random values that are difficult to foresee (similarly to observations errors). In practice, it is thus impossible to eliminate their influence on the vector of differential observations $\delta_{y}$, and thus it is impossible to actually determine the ${\hat{u}}_{p}$ estimator. Extending the observation model to the form of Equation (24) and applying the proposed TLSC method lead to different solutions. In this case, the calculation results can go beyond TLSC estimators of the **u** deterministic displacements, but also include estimations of the $\overset{⌢}{\eta }$ random displacements (with their prediction for ECPs), and estimations of the $\overset{⌢}{E}$ noise matrices. The example of this solution is presented below.

The example assumes the same simulated FCN as above. The simulation was carried out for the following vector of CPs’ theoretical displacements of $\bar{u}={\left[{\bar{u}}_{1}^{},\;{\bar{u}}_{2}^{},\;{\bar{u}}_{3}^{},\;\cdots ,\;{\bar{u}}_{25}^{}\right]}^{T}={\left[-5,\;0,\;0,\;\cdots \;,\;0\right]}^{T}{}_{\mathrm{mm}}$. The following values $\sigma_{v}=0.3\;\mathrm{mm}$, $\sigma_{s}=1.0\;\mathrm{mm}$, and $\sigma_{E}=0.1\;\mathrm{mm}$ were adopted for each of the measurement epochs $t_{\alpha }$ and $t_{\beta }$. Similarly to the previous analysis, the correlation coefficient between the signals in the most distant points is $\rho_{\min}=0.01$. The vector of observations differences $\delta_{y,sim}^{}=A\bar{u}+\xi_{sim}^{}$, where $\xi_{sim}=(B+{\overset{⌢}{E}}_{sim}){\overset{⌢}{\eta }}_{sim}+\upsilon_{sim}$, was determined in the same way. The vector was the basis for iterative calculations performed with the use of algorithm defined by Equations (51)–(58). The starting matrix of random noise ${\overset{⌢}{E}}^{\left(0\right)}$ was generated by adopting $\sigma_{E^{\left(0\right)}}=0.01,\;\sigma_{E}=0.001\;\mathrm{mm}$. The objects of these calculations are as follows: the $\hat{u}$ estimator of CPs’ deterministic displacements, the $\hat{\eta }$ estimation of these points’ random displacements, the $\hat{\eta^{\prime }}$ prediction of ECPs random displacements, the total CPs displacement ${\hat{u}}_{\eta }=\hat{u}+\hat{\eta }$, prediction ${{\hat{u}}^{\prime }}_{\eta }=\hat{u^{\prime }}+{\hat{\eta }}^{\prime }$ of ECPs’ total displacements, and estimation $\hat{\overset{⌢}{E}}$ of the noise matrix. By comparison, the classic least squares estimator ${\hat{u}}_{LS}$ of the **u** parameter was also determined. Model $\delta_{y}=Au+\upsilon$ was the basis for determining estimator ${\hat{u}}_{LS}$ and was used for the same observations’ differences vector $\delta_{y,sim}$ as above. Simulated and determined values are presented in Table 2. The table also presents the values of the following comparative parameters

(62) $$\begin{array}{c} J_{\hat{u}}={\left({\left\|\bar{u}-\hat{u}\right\|}^{2}/r\right)}^{1/2},J_{{\hat{u}}_{LS}}={\left({\left\|\bar{u}-{\hat{u}}_{LS}\right\|}^{2}/r\right)}^{1/2},\\ J_{\hat{\eta }}={\left({\left\|\eta -\eta \right\|}^{2}/r\right)}^{1/2},J_{{\hat{u}}_{\eta }}={\left({\left\|\left({\bar{u}}_{\eta }-{\hat{u}}_{\eta }\right)\right\|}^{2}/r\right)}^{1/2} \end{array}$$

where ${\bar{u}}_{\eta }=\bar{u}+\eta_{sim}$ is the simulated total displacement.

**Figure 3:**
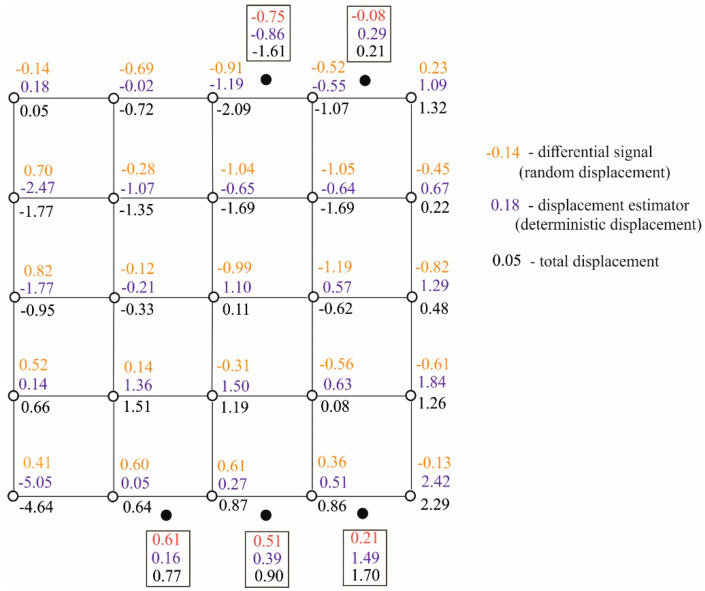
Random, deterministic, and total displacements of points within the simulated free control network (in mm).

The random displacements’ prediction ${\hat{\eta }}^{\prime }$ allows for prediction of ECPs’ total displacements. However, an interpolation of deterministic displacements $u^{\prime }$ of these points must be carried out first. Assume that point $P_{i}^{\prime }\in P^{\prime }$ lies between points $P_{k}P_{l}\in P$, and for these points, the displacements estimators ${\hat{u}}_{k}$ and ${\hat{u}}_{l}$ have been determined. Then, in the simplest case, the linear interpolation expressed by the following formula can be used to determine the displacement ${\hat{u^{\prime }}}_{i}$

(63) $${{\hat{u}}^{\prime }}_{i}={\hat{u}}_{k}+\frac{{\hat{u}}_{l}-{\hat{u}}_{k}}{d_{k,l}}d_{k,i}$$

where $d_{k,l}$ is the distance between points $P_{k}$ and $P_{l}$, whereas $d_{k,i}$ is the distance between point $P_{k}$ and point $P_{i}{}^{\prime }$. Interpolated displacements of points $P_{1}{}^{\prime },\ldots P_{5}{}^{\prime }$ (the ${\hat{u}}^{\prime }$ vector) and total displacements of these points (vector ${{\hat{u}}^{\prime }}_{\eta }={\hat{u}}^{\prime }+{\hat{\eta }}^{\prime }$) determined using Equation (53) are also presented in Table 2. Final information about random, deterministic, and total displacements of all points (CPs and ECPs) within a simulated free control network is presented in Figure 3.

**Figure 4:**
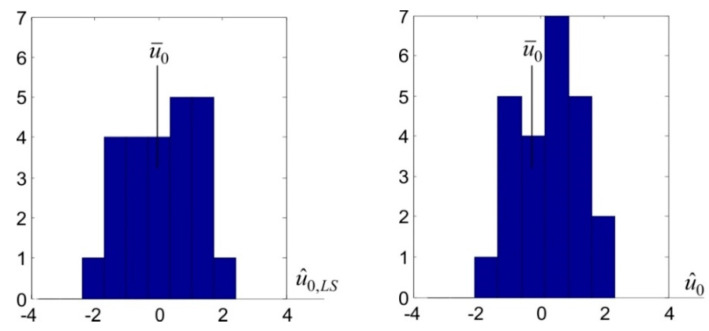
The histograms of least squares (LS) and total least-squares collocation (TLSC) estimators regarding zero CPs displacements.

While analysing the obtained results, attention should be paid to the values of comparative parameters $J_{{\hat{u}}_{LS}}=0.92\;\mathrm{mm}$ and $J_{\hat{u}}=0.79\;\mathrm{mm}$. These values mean that the deterministic displacement estimators designated using this method are generally closer to the theoretical values of these displacements, compared with corresponding LS estimators. This particularly applies to displacement $u_{1}$, for which the absolute values of differences between its relatively high theoretical value ${\bar{u}}_{1}=-5\;\mathrm{mm}$ and the designated estimators are $\left|{\bar{u}}_{1}-{\hat{u}}_{1,LS}\right|=0.57\;\mathrm{mm}$ and $\left|{\bar{u}}_{1}-{\hat{u}}_{1}\right|=0.05\;\mathrm{mm}$, respectively. The theoretical displacements of the remaining points are equal to zero. Assume that such displacements are the $u_{0}$ parameter that is common to these points. Then, LS and TLSC estimators of parameter $u_{0}$ can be treated as the realisations ${\hat{u}}_{0,k,LS}$ and ${\hat{u}}_{0,k}$, k=1,…,25 of two random variables, ${\hat{u}}_{0,LS}$ and ${\hat{u}}_{0}$, respectively. In this approach, the comparisons regarding the quality of identification of zero displacement can be made on the basis of the determined estimators’ histograms (Figure 4). These histograms indicate that, compared with LS estimators, TLSC estimators are more focused around the theoretical value ${\bar{u}}_{0}=0\;\mathrm{mm}$.

**Figure 5:**
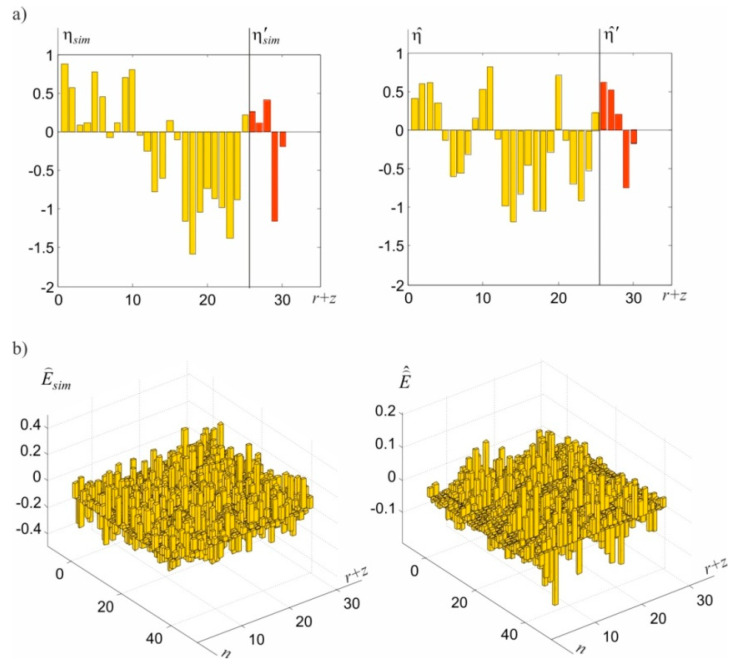
Graphic illustration of estimation results: (**a**) simulated and determined random displacements of CPs and ECPs, (**b**) simulated and determined surface noise in these points.

Taking into account the issue of determining the network points’ displacements, it is interesting that total displacement estimators agree with their simulated values. This is displayed by parameter $J_{{\hat{u}}_{\eta }}=0.55\;\mathrm{mm}$, which differs insignificantly from parameter $J_{\hat{\eta }}=0.62\;\mathrm{mm}$ that defines the quality of random displacements estimation. These estimations are generally similar to the simulated values (Figure 5a), whereas this similarity is also applicable for predictions at ECPs.

**Figure 6:**
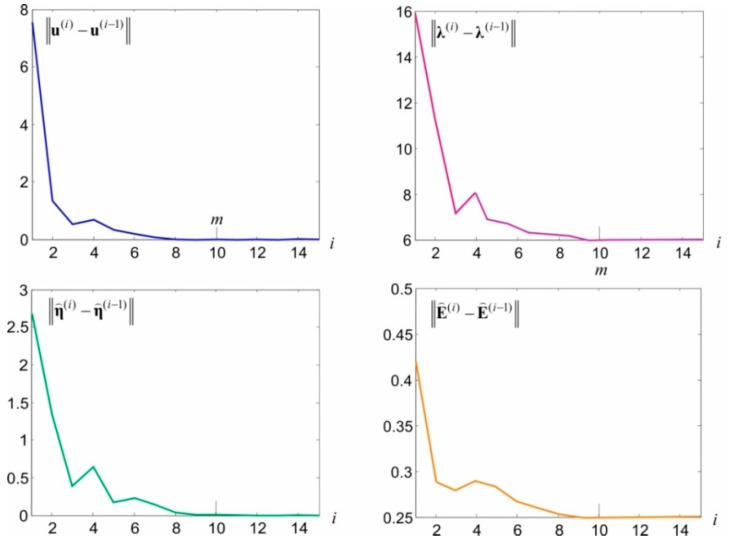
The course of controlled values of norms in individual iteration steps.

Owing to the random character of theoretical signals, a better match can hardly be expected here, particularly with reference to specific, individual signals. Attention should also be paid to the random noise estimations at CPs and ECPs, presented in Figure 5b. These general estimations have lower values compared with simulated noise. This is primarily caused by the adopted, relatively small standard deviation of these values. In this situation, although the adopted model should prevent them, some noise becomes part of observations errors. The consequence of this autonomous integration is that the assessments of the $\overset{⌢}{E}$ matrix elements are underestimated. From the theoretical point of view, this could be prevented by adopting a higher value for $\sigma_{E}$, for example, comparable with the value of the observation’s standard deviation. The conducted numerical tests have, however, indicated that the iteration under Equations (51)–(58) is not convergent in many cases. Substituting the proposed, convenient iteration procedure with a different procedure in relation to such cases is an important issue, which is, however, beyond the scope of this paper. In the presented example, the iteration process for end criteria $\xi_{u}=\xi_{\eta }=\xi_{E}=0.001\;\mathrm{mm}$ and $\xi_{\lambda }=0.001\;\mathrm{mm}$ ended after m=10 steps. The values of norms $\left\|u^{\left(i\right)}-u^{\left(i-1\right)}\right\|$, $\left\|\lambda^{\left(i\right)}-\lambda^{\left(i-1\right)}\right\|$, $\left\|{\overset{⌢}{\eta }}^{\left(i\right)}-{\overset{⌢}{\eta }}^{\left(i-1\right)}\right\|$, and $\left\|{\overset{⌢}{E}}^{\left(i\right)}-{\overset{⌢}{E}}^{\left(i-1\right)}\right\|$ obtained in individual steps are presented in Figure 6.

### 4.2. Real Free Control Network

**Figure 7:**
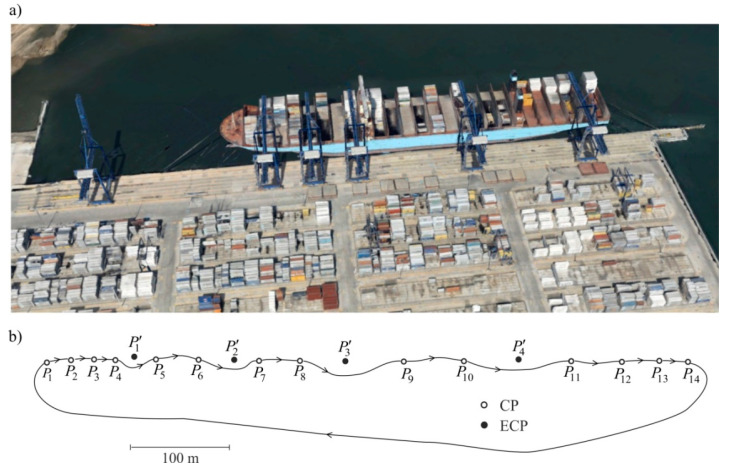
Monitored loading quay: (**a**) object’s photo (source: Google Maps), (**b**) free control network (FCN) diagram with ECPs marked.

The simulations presented in the previous section have been supplemented with the results of real FCN calculations. The network was established to monitor the loading quays (Figure 7a). It is comprised of CPs $P_{1},\ldots ,P_{14}$, among which heights differences were measured (Figure 7b). In difficult to measure but critical points of the quay, points ${P^{\prime }}_{1},\ldots ,{P^{\prime }}_{4}$ were created, to be treated as ECPs.

The calculations were performed for two measurement epochs: the year $t_{\alpha }=1998$ and the year $t_{\beta }=2008$. The specific nature of operations carried out on port quays justifies the application of the proposed method. The ongoing operation of gantries and other loading equipment, the stacking and lifting containers, and the docking of vessels can cause vibrations and elastic displacements. In this case, not only the object’s deterministic deformations, but also random fluctuations consisting of signals and surface noise can be expected.

**Figure 8:**
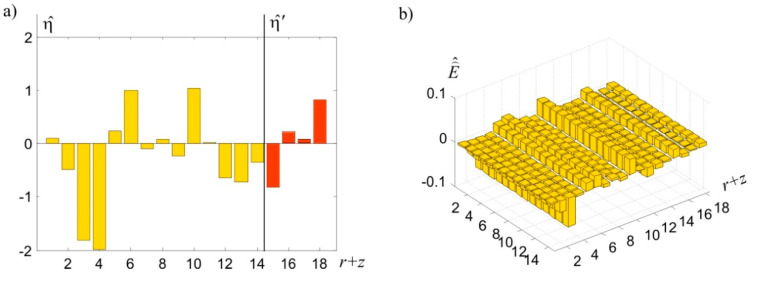
Graphic illustration of (**a**) random displacement and (**b**) surface noise.

**Figure 9:**
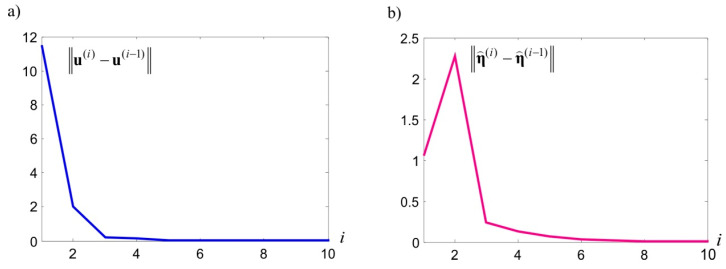
The courses of norms values in the iterative process: (**a**) random displacement (**b**) surface noise.

Observations of heights differences $h_{\left(k,l\right)}$ among points $P_{k},\;P_{l}$, k=1,…,14, l=2,…,14,1 and differences $\delta_{y_{i}}=h_{(k,l)\beta }-h_{(k,l)\alpha }$, i=(k,l) among these heights differences, obtained in both measurement epochs, are presented in Table 3. In each measurement epoch, observations were obtained assuming standard deviation $\sigma_{v}=0.2\;\mathrm{mm}$. In practice, the selection of adequate signals values and surface noise’s standard deviation is an important issue. In this example, calculations were performed in two versions: for signal’s standard deviation, clearly exceeding observations’ standard deviation ($\sigma_{s}=3\sigma_{\nu }=0.6\;\mathrm{mm}$), and when these standard deviations are equal to each other ($\sigma_{s}=\sigma_{\nu }=0.2\;\mathrm{mm}$). In both versions, identical standard deviations were adopted for surface noise $\sigma_{E}=\;0.1\;\mathrm{mm}$. The subject of the calculations was the deterministic, random, and total CPs’ displacements (vectors $\hat{u}$, $\hat{\eta }$, and ${\hat{u}}_{\eta }=\hat{u}+\hat{\eta }$, respectively). For comparative reasons, a classic estimator of displacement ${\hat{u}}_{LS}$ for these points was also determined. The calculations also produced the prediction ${\hat{\eta }}^{\prime }$ of random displacements regarding ECPs (i.e., points $P_{1}{}^{\prime },\ldots ,P_{4}{}^{\prime }$). The ${\hat{\eta }}^{\prime }$ vector and interpolated displacements ${\hat{u}}^{\prime }$ of ECPs were then used to determine their total displacements ${\hat{u}}_{\eta }=\hat{u}+\hat{\eta }$. The results are shown in Table 4. A graphic presentation of determined random displacements and surface noise is presented in Figure 8a,b. The iteration process leading to the presented results ends after eight steps. The course of the most important norms $\left\|u^{\left(i\right)}-u^{\left(i-1\right)}\right\|$ and $\left\|{\overset{⌢}{\eta }}^{\left(i\right)}-{\overset{⌢}{\eta }}^{\left(i-1\right)}\right\|$ essential to this process is presented in Figure 9.

The TLSC estimators of deterministic displacements for both versions of signals’ standard deviations vary from each other. This is shown by the ${\left({\left\|{\hat{u}}_{0.6}-{\hat{u}}_{0.2}\right\|}^{2}/r\right)}^{1/2}=0.55\;\mathrm{mm}$ parameter, where ${\hat{u}}_{0.2}$ and ${\hat{u}}_{0.6}$ are heights’ difference estimators obtained for signals’ standard deviations, $\sigma_{s}=0.2\;\mathrm{mm}$ and $\sigma_{s}=0.6\;\mathrm{mm}$, respectively. The estimations of random displacements, for which ${\left({\left\|{\hat{\eta }}_{0.6}-{\hat{\eta }}_{0.2}\right\|}^{2}/r\right)}^{1/2}=0.58\;\mathrm{mm}$ is obtained, vary in a similar way. With reference to real FCNs, it is particularly important to compare TLSC estimators of deterministic displacements with their classic LS estimators. Calculating relevant comparative indicators, the results are as follows:${\left({\left\|{\hat{u}}_{0.2}-{\hat{u}}_{LS}\right\|}^{2}/r\right)}^{1/2}=0.28\;\mathrm{mm}$ and ${\left({\left\|{\hat{u}}_{0.6}-{\hat{u}}_{LS}\right\|}^{2}/r\right)}^{1/2}=0.83\;\mathrm{mm}$. For the variant where $\sigma_{s}=0.6\;\mathrm{mm}$, the difference between TLSC and LS estimators of deterministic displacements can have a practical significance. However, paying attention to the total displacements of the analysed network points, for these displacements, where $\sigma_{s}=0.6\;\mathrm{mm}$ ${\left({\left\|{\hat{u}}_{\eta ,0.6}-{\hat{u}}_{LS}\right\|}^{2}/r\right)}^{1/2}=0.28\;\mathrm{mm}$ is obtained. This parameter shows that the total displacement estimations obtained using the TLSC method hardly differ from the LS estimators of deterministic displacements (for $\sigma_{s}=0.2\;\mathrm{mm}$, this parameter is 0.09 mm). This means that the displacements that were treated as deterministic in the LS method have been split into deterministic and random in the TLSC method.

Additionally, in this practical example, the prediction of ECPs’ random displacements (for the version in which $\sigma_{s}=0.6\;\mathrm{mm}$) was analysed. For instance, point ${P^{\prime }}_{1}$ lies between points $P_{4}$ and $P_{5}$ at similar distances, $d_{{P^{\prime }}_{1}-P_{4}}=23\;m$ and $d_{{P^{\prime }}_{1}-P_{5}}=26\;m$. The random displacements recorded for these points are ${\hat{\eta }}_{P_{4}}=-1.99\;\mathrm{mm}$ and ${\hat{\eta }}_{P_{5}}=0.24\;\mathrm{mm}$. At point ${P^{\prime }}_{1}$, the random displacement prediction determined using the TLSC method is ${{\hat{\eta }}^{\prime }}_{{P^{\prime }}_{1}}=-0.83\;\mathrm{mm}$, which is the approximate average of the displacements recorded in proximate points (this average is −0.875 mm). At this moment, it should be noted that the closer CP has a bigger (but still unclear) influence on the random displacement’s prediction at ECP. A different ECP, that is, point ${P^{\prime }}_{4}$, lies at distances from points $P_{10}$ and $P_{11}$ of $d_{{P^{\prime }}_{4}-P_{10}}=35\;m$ and $d_{{P^{\prime }}_{4}-P_{11}}=69\;m$. For these points, the assessments of random vertical displacements are ${\hat{\eta }}_{P_{10}}=1.03\;\mathrm{mm}$ and ${\hat{\eta }}_{P_{11}}=0.02\;\mathrm{mm}$. The determined prediction of displacement of point ${P^{\prime }}_{4}$ is equal to ${{\hat{\eta }}^{\prime }}_{{P^{\prime }}_{4}}=0.81\;\mathrm{mm}$. In this case, the impact of closer CP on the displacement prediction at ECP is definitely larger.

## 5. Conclusions

In this paper, the free control network was limited to the class of levelling networks. Such networks play the important role in vertical surface deformations analyses. Splitting the points’ displacements within an FCN into deterministic and random parts allows for expansion of the analysis, primarily in terms of the character of monitored surface deformations. Identification of this aspect can be of high practical importance. The different object’s reactions to deterministic deformations as well as to random surface fluctuations can be expected. There are different ways of interpreting these deformations and methods of preventing their effects.

The total displacement estimations obtained using the TLSC method hardly differ from the LS estimators of deterministic displacements. When these methods are applied, the general conclusions pertaining to network deformations can be similar. Therefore, if the character of deformations is not the object of interest, TLSC may be treated as an alternative to the LS method. The proposed method, however, opens the path to more detailed interpretation of displacements, and to these magnitudes’ prediction, for additional points of the monitored surface that are not connected to the control network.

The solution of the optimisation problem in TLSC is iterative. The iterative process is generally convergent and ended after 8–10 steps in the tests conducted as part of the study. The problem with the convergence of the iterative process occurs only when the surface noise’s standard deviations are too large (equal to or exceeding the values of observations’ standard deviations). Selecting the surface noise’s standard deviations, adequate to the specific, monitored object, as well as signals’ standard deviations, is a separate problem requiring additional analyses. This also applies to the selection of adequate covariance functions and their parameters. In this study, the role of empirical examples and analyses was limited to demonstrating the results that could be expected after applying the proposed method. For this reason, the most popular Gaussian’s covariance function using the parameter coming from the adopted, minimum value of correlation coefficient was applied. In specific instances, this function can be replaced by a different, more justified covariance function.

## Figures and Tables

**Table 1:** Comparative parameters $J_{\hat{u}}^{MC}$ and $J_{{\hat{u}}_{p}}^{MC}$ obtained with the Monte Carlo (MC) method.

$\sigma_{s}=0$												
	${\bar{u}}_{1}=0$				${\bar{u}}_{1}=-5$				${\bar{u}}_{1}=-10$			
	$\sigma_{E}$				$\sigma_{E}$				$\sigma_{E}$			
	0.0	0.1	0.2	0.3	0.0	0.1	0.2	0.3	0.0	0.1	0.2	0.3
$J_{\hat{u}}^{MC}$	0.24	0.24	0.24	0.24	0.29	0.29	0.29	0.29	0.40	0.40	0.40	0.40
$J_{{\hat{u}}_{p}}^{MC}$	0.24	0.24	0.24	0.24	0.29	0.29	0.29	0.29	0.40	0.40	0.40	0.40
$\sigma_{s}=0.5$												
	${\bar{u}}_{1}=0$				${\bar{u}}_{1}=-5$				${\bar{u}}_{1}=-10$			
	$\sigma_{E}$				$\sigma_{E}$				$\sigma_{E}$			
	0.0	0.1	0.2	0.3	0.0	0.1	0.2	0.3	0.0	0.1	0.2	0.3
$J_{\hat{u}}^{MC}$	0.46	0.63	0.99	1.41	0.49	0.66	1.00	1.40	0.57	0.71	1.03	1.44
$J_{{\hat{u}}_{p}}^{MC}$	0.24	0.24	0.24	0.24	0.29	0.29	0.29	0.29	0.40	0.40	0.40	0.40
$\sigma_{s}=1.0$												
	${\bar{u}}_{1}=0$				${\bar{u}}_{1}=-5$				${\bar{u}}_{1}=-10$			
	$\sigma_{E}$				$\sigma_{E}$				$\sigma_{E}$			
	0.0	0.1	0.2	0.3	0.0	0.1	0.2	0.3	0.0	0.1	0.2	0.3
$J_{\hat{u}}^{MC}$	0.81	0.93	1.21	1.55	0.84	0.94	1.20	1.56	0.89	1.00	1.24	1.58
$J_{{\hat{u}}_{p}}^{MC}$	0.24	0.24	0.24	0.24	0.29	0.29	0.29	0.29	0.40	0.40	0.40	0.40

**Table 2:** The results of estimation and prediction (in mm) for the simulated free control network (FCN).

CP	$\bar{u}$	${\hat{u}}_{LS}$	$\hat{u}$	$\eta_{sim}$	$\hat{\eta }$	${\bar{u}}_{\eta }=\bar{u}+\eta_{sim}$	${\hat{u}}_{\eta }=\hat{u}+\hat{\eta }$
$P_{1}$	−5	−4.43	−5.05	0.87	0.41	−4.13	−4.64
$P_{2}$	0	0.86	0.05	0.57	0.60	0.57	0.64
$P_{3}$	0	1.09	0.27	0.09	0.61	0.09	0.87
$P_{4}$	0	1.08	0.51	0.11	0.36	0.11	0.86
$P_{5}$	0	2.50	2.42	0.78	−0.13	0.78	2.29
$P_{6}$	0	1.44	1.84	0.45	−0.61	0.45	1.23
$P_{7}$	0	0.29	0.63	−0.07	−0.56	−0.07	0.08
$P_{8}$	0	1.40	1.50	0.11	−0.31	0.11	1.19
$P_{9}$	0	1.72	1.36	0.71	0.14	0.71	1.51
$P_{10}$	0	0.88	0.14	0.81	0.52	0.81	0.66
$P_{11}$	0	−0.73	−1.77	−0.05	0.82	−0.05	−0.95
$P_{12}$	0	−0.11	−0.21	−0.26	−0.12	−0.26	−0.33
$P_{13}$	0	0.33	1.10	−0.78	−0.99	−0.78	0.11
$P_{14}$	0	−0.40	0.57	−0.61	−1.19	−0.61	−0.62
$P_{15}$	0	0.69	1.29	0.14	−0.82	0.14	0.48
$P_{16}$	0	0.44	0.67	−0.11	−0.45	−0.11	0.22
$P_{17}$	0	−1.48	−0.64	−1.15	−1.05	−1.15	−1.69
$P_{18}$	0	−1.47	−0.65	−1.58	−1.04	−1.58	−1.69
$P_{19}$	0	−1.13	−1.07	−1.04	−0.28	−1.04	−1.35
$P_{20}$	0	−1.55	−0.47	−0.73	0.70	−0.73	−1.77
$P_{21}$	0	0.27	0.18	−0.86	−0.14	−0.86	0.05
$P_{22}$	0	−0.50	−0.02	−0.98	−0.69	−0.98	−0.72
$P_{23}$	0	−1.88	–1.19	–1.38	–0.91	–1.38	–2.09
$P_{24}$	0	–0.85	– 0.55	–0.87	–0.52	–0.87	–1.07
$P_{25}$	0	1.53	1.09	0.22	0.23	0.22	1.32
	$J_{{\hat{u}}_{LS}}=0.92$ $J_{\hat{u}}=0.79$			$J_{\hat{\eta }}=0.62$		$J_{{\hat{u}}_{\eta }}=0.55$	
ECP			${\hat{u}}^{\prime }$	${\eta^{\prime }}_{sim}$	$\hat{\eta^{\prime }}$		${{\hat{u}}^{\prime }}_{\eta }={\hat{u}}^{\prime }+{\hat{\eta }}^{\prime }$
${P^{\prime }}_{1}$	–	–	0.16	0.19	0.61	–	0.77
${P^{\prime }}_{2}$	–	–	0.39	0.10	0.51	–	0.90
${P^{\prime }}_{3}$	–	–	1.49	0.41	0.21	−	1.70
${P^{\prime }}_{4}$	−	−	−0.86	−1.16	−0.75	−	−1.61
${P^{\prime }}_{5}$	−	−	0.29	−0.19	−0.08	−	0.21

**Table 3:** Observation of heights’ differences within a real FCN (in mm).

	$h_{\left(1,2\right)}$	$h_{\left(2,3\right)}$	$h_{\left(3,4\right)}$	$h_{\left(4,5\right)}$	$h_{\left(5,6\right)}$	$h_{\left(6,7\right)}$	$h_{\left(7,8\right)}$	$h_{\left(8,9\right)}$	$h_{\left(9,10\right)}$	$h_{\left(10,11\right)}$	$h_{\left(11,12\right)}$	$h_{\left(12,13\right)}$	$h_{\left(13,14\right)}$	$h_{\left(14,1\right)}$
$t_{\alpha }$	−6.9	11.6	−13.2	−25.3	−3.6	−14.9	20.6	−3.4	8.5	−15.7	8.2	0.1	12.1	−29.7
$t_{\beta }$	−3.5	8.9	−6.6	23.4	−1.2	−13.9	23.2	−2.7	10.8	−17.5	7.9	−0.4	14.8	−38.2
$\delta_{y_{i}}$	3.4	−2.7	6.6	−1.9	2.4	1.0	2.6	0.7	2.3	−1.8	−0.3	−0.5	2.7	−8.5

**Table 4:** The results of estimation and prediction for the real FCN (in mm).

**CP**	${\hat{u}}_{LS}$	$\hat{u}$		$\hat{\eta }$		${\hat{u}}_{\eta }=\hat{u}+\hat{\eta }$	
		$\sigma_{s}=0.2$	$\sigma_{s}=0.6$	$\sigma_{s}=0.2$	$\sigma_{s}=0.6$	$\sigma_{s}=0.2$	$\sigma_{s}=0.6$
$P_{1}$	−5.97	−6.10	−6.35	0.03	0.10	−6.07	−6.24
$P_{2}$	−2.98	−2.91	−2.76	−0.16	−0.49	−3.07	−3.25
$P_{3}$	−6.12	−5.60	−4.58	−0.61	−1.82	−6.21	−6.39
$P_{4}$	0.05	0.62	1.76	−0.66	−1.99	−0.04	−0.23
$P_{5}$	−2.27	−2.44	−2.78	0.08	0.24	−2.36	−2.54
$P_{6}$	−0.29	−0.72	−1.57	0.33	1.00	−0.39	−0.57
$P_{7}$	0.27	0.21	0.09	−0.03	−0.09	0.18	−0.00
$P_{8}$	2.45	2.33	2.09	0.03	0.08	2.36	2.17
$P_{9}$	2.72	2.70	2.67	−0.08	−0.23	2.62	2.44
$P_{10}$	4.60	4.16	3.30	0.34	1.03	4.50	4.32
$P_{11}$	2.35	2.25	2.06	0.01	0.02	2.26	2.07
$P_{12}$	1.59	1.72	1.96	−0.21	−0.64	1.51	1.32
$P_{13}$	0.66	0.80	1.09	−0.24	−0.71	0.56	0.38
$P_{14}$	2.95	2.97	3.02	−0.12	−0.35	2.85	2.67
ECP		${\hat{u}}^{\prime }$		$\hat{\eta^{\prime }}$		${{\hat{u}}^{\prime }}_{\eta }={\hat{u}}^{\prime }+{\hat{\eta }}^{\prime }$	
${P^{\prime }}_{1}$		−0.81	−0.37	−0.28	−0.83	−1.09	−1.20
${P^{\prime }}_{2}$		−0.13	−0.51	0.07	0.21	−0.06	−0.30
${P^{\prime }}_{3}$		2.48	2.32	0.01	0.04	2.49	2.36
${P^{\prime }}_{4}$		3.52	2.87	0.27	0.81	3.79	3.68
